# Vitamin D Receptor Gene Polymorphisms Influence T1D Susceptibility among Pakistanis

**DOI:** 10.1155/2017/4171254

**Published:** 2017-12-03

**Authors:** Maryam Mukhtar, Andleeb Batool, Abdul Wajid, Iram Qayyum

**Affiliations:** ^1^Department of Zoology, G.C. University, Punjab, Lahore 54000, Pakistan; ^2^Department of Biotechnology, Virtual University of Pakistan, 1-Davis Road Lahore 54000, Pakistan

## Abstract

**Background:**

The vitamin D receptor (*VDR*) gene regulates insulin secretion from the pancreas and acts as a mediator of the immune response through vitamin D. Polymorphism in *VDR* causes alterations in the functioning of vitamin D, leading to type 1 diabetes (T1D) predisposition. The aim of the present study was to determine *VDR* gene polymorphism in association with T1D in Pakistanis.

**Methods:**

The association was evaluated by selecting rs2228570 (*FokΙ*), rs7975232 (*Apa*Ι), and rs731236 (*Taq*Ι) polymorphic sites in 102 patients and 100 controls. Genotypes were identified by DNA sequencing and PCR-RFLP.

**Results:**

The allelic and genotypic frequencies of *FokΙ* and *Apa*I were significantly associated with T1D (*p* < 0.001) development. At the *Fok*Ι site, tryptophan was replaced with arginine due to polymorphism. A novel SNP (GeneBank acc number KT280406) was identified through the sequencing of intron 8, 62 bp downstream from the *Apa*I polymorphic site, and significantly associated with T1D development. The *TaqΙ* did not depict any association with T1D at the allelic or genotypic level (*p* > 0.05). CCGC, CCGG, CCTC, and CCTG haplotypes were significantly associated with disease development (*p* < 0.05). However, CTGG haplotype was protective towards T1D (*p* < 0.01).

**Conclusion:**

*VDR* polymorphisms were identified as susceptible regions for T1D development in the Pakistani population.

## 1. Introduction

T1D is a polygenic disease with several protective and susceptible alleles interacting with each other [1]. Genetics plays a key role in the onset of T1D [2, 3] and shows a significant clustering in a family; as in siblings, the average risk prevalence is 6% as compared to the general population with a 0.6% risk [4].

Vitamin D displays vital immunomodulatory properties that aid in preventing diabetes development in T1D animal models [5]. It activates human macrophages, antigen-presenting cell maturation, and inhibits dendritic cell differentiation as well as affects cytokine production by interacting with most immune cells [6, 7]. Vitamin D reduced the MHC class I molecules and Fas expression (transmembrane cell surface receptor mediator) leading to the suppression of pancreatic *β*-cell apoptosis [8, 9]. In addition, it increases A20 protein expression, which has an antiapoptotic function against pancreatic *β*-cells, and increases insulin production [9].

Vitamin D is reported to be metabolically active and activates the nuclear vitamin D receptors (*VDR*) to exert its genomic effect. The *VDR* belongs to the super family of ligand-activated transcription factors located on the 12q12–q14 chromosome and encoded by the *VDR* gene in humans [10]. It consists of two promoter regions, six untranslated exons (exons 1a–1f) that are spliced alternatively, and eight protein-coding exons (exons 2–9) [11]. Basically, the *VDR* gene contains four polymorphism sites which were identified with restriction fragment length polymorphism (RFLPs) *Taq*I within exon 9, *ApaΙ* and BsmI within intron 8, *Fok*I within exon 2, and mononucleotide polymorphism in the 3′ untranslated region [12]. In our previous study, two SNPs: rs1544410 on *VDR* and rs2476601 on *PTPN22* were screened and were found significantly associated with T1D in the Pakistani population [13]. The genomic action of vitamin D is initiated by its binding to the *VDR*, which leads to the transcription of genes regulated by it. This transcriptional regulation occurs to a very complicated mechanism [14].

The Pakistani population is heterogeneous and becoming more complex due to cast-specific marriages. This phenomenon produces narrow genetic pool and transfers the genetic mutations more often to the next generations. Therefore, the current study was designed to evaluate the association of polymorphism in the *VDR* gene with T1D.

## 2. Methods

### 2.1. Subjects

The study was ethically approved by the Board of Advance Research, G.C. University, Lahore, Pakistan. This case-control study was carried on T1D patients recruited from the Diabetic Center of Shalamar Hospital, Lahore (public sector hospital). Written consent was obtained from patients/guardians of the studied subjects. All patients that participated were already clinically diagnosed with T1D by a physician according to WHO criteria such as hyperglycemia, insulin requirement from diagnosis, recurrent infections, high levels of glycosuria, increased urine volume and thirst, unexplained weight loss, and in severe cases coma and drowsiness [15]. A total of 102 T1D cases and 100 controls were included in the study. The clinical characteristics (gender, age, the age of diagnosis, and positive family history as well as physical activity including exercise and playing outdoor games) that were recorded after interviewing the patients who participated in the study were presented in Table 1. All control subjects were healthy and had a negative family history of T1D.

### 2.2. DNA Isolation and SNP Selection

Blood samples (3 ml) from each subject were collected in EDTA-coated tubes, and DNA was extracted by the modified organic extraction method [16]. The extracted DNA was stored at −20°C (Haier) for further genetic analysis. DNA quantification was carried out by the nanodrop (Thermo 2000). Three polymorphic sites *Fok*I, *Apa*I, and *Taq*I were selected by using the HapMap database (http://hapmap.ncbi.nlm.nih.gov/) and SNP Browser software 4.0 (Applied Biosystems).

### 2.3. Polymerase Chain Reaction

The DNA was amplified for polymerase chain reaction (PCR) in a 25 *μ*l reaction mixture by using already reported primers (Israni et al., [17]). The following primers were used: for *Fok*I (rs2228570) forward primer (F): 5′-AGCTGGCCCTGGCACTGACTCTGGCTCT-3′ and reverse primer (R): 3′-ATGGAAACACCTTGCTTCTTCTCCCTC-5′, for *Taq*I (rs731236), *Apa*I (rs7975232), and KT280406 F: 5′-CAGAGCATGGACAGGGAGCAA-3′ and R: 3′-GCAACTCCTCATGGCTGAGGTCTC-5′. The PCR was carried out for 30 cycles, which consists of initial denaturation at 94°C for 5 min, denaturation at 94°C for 45 s, annealing at 68°C (*Fok*I) and 65°C (*Taq*I and *Apa*I) for 45 s, and extension at 72°C for 30 s followed by final extension at 72°C for 10 mins.

### 2.4. *VDR* Genotyping

*VDR* genotyping for *Fok*I, *Apa*I, and *Taq*I was performed by DNA sequencing and restriction fragment length polymorphism (RFLP), whereas for KT280406, all samples were sequenced with forward primer. For sequencing, the reaction mixture of 10 *μ*l was prepared to contain 3 *μ*l of purified PCR product, 1 *μ*l of forward primer, 1 *μ*l of big dye mixture, 1 *μ*l of 5X reaction buffer, and 4 *μ*l of DEPC water. The products were amplified by following PCR conditions: initial denaturation at 95°C for 2 mins followed by repeated 35 cycles of denaturation at 95°C for 30 s, annealing at 50°C for 15 s, and extension at 60°C for 4 mins, and at end final extension at 60°C for 5 mins. By using isopropanol, amplified PCR products were precipitated. Product pellets were dissolved in 12 *μ*l formamide and were incubated for 5 minutes at 95°C and chilled quickly. The samples were loaded to ABI PRISM genetic analyzer 3130 XL to sequence the fragment of interest. The samples were visualized by sequencing software v 3.7 and Bio Edit software, and mutations were determined (Supplementary Figures 1 available online at https://doi.org/10.1155/2017/4171254, 2, and 3) and were confirmed by NCBI BLAST (Supplementary Figure 4).

**Figure 1:**
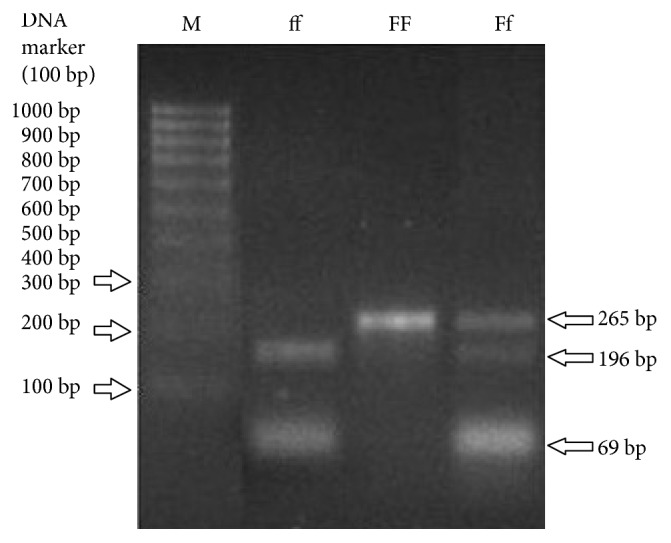
*FokΙ* digestion (SNP C/T) in exon 2: Restriction site presence is designated by “f,” and absence is designated by “F.” ff (CC), for example, a 196 bp and 69 bp; Ff (CT), for example, 265 bp, 196 bp, and 69 bp, bands; FF (TT), for example, 196 bp and 69 bp bands.

For PCR-RFLP, according to the manufacturer's instructions, the PCR products were digested using restriction enzymes; BseGI (*Fok*I) (Fermentas, Germany), *Apa*I, and *Taq*I (Vivantis). Briefly, 10 *μ*l of each PCR product was mixed with 2 *μ*l of Tango buffer, 1 *μ*l of restriction enzyme, and 12 *μ*l of DEPC water. The tubes were incubated at 55*°*C for 5 h (*Fok*I), 55°C for 3 h (*Taq*I), and at 37°C for 16 h (*Apa*I); followed by thermal inactivation of restriction enzymes at 80°C (*Fok*I), 80°C (*Taq*I), and 65°C (*Apa*I) for 20 mins. Digested samples were run on 2% agarose gel and visualized on the gel documentation system (BioDoc-It Imaging System, Figure 1).

### 2.5. Statistical Analysis

All data of the controls passed the Hardy-Weinberg equilibrium (*p* > 0.05). The chi-square test was used to determine allelic and genotypic frequencies. Linkage disequilibrium and haplotypes were calculated to study their association with T1D by SHEsis (http://analysis.bio-x.cn/SHEsisMain.htm). The change in amino acid sequences was determined by aligning sequences in Mega 6 software.

## 3. Results

The allelic and genotype frequencies of the *VDR* gene at *Fok*I, *Apa*I, and *Taq*I polymorphic sites were assessed in 102 T1D patients and 100 controls and were presented in Table 2. In patients and controls, *Fok*I, *Apa*I, *Taq*I, and KT280406 were validated (*p* > 0.05) by the Hardy-Weinberg equilibrium (HWE).

**Figure 2:**
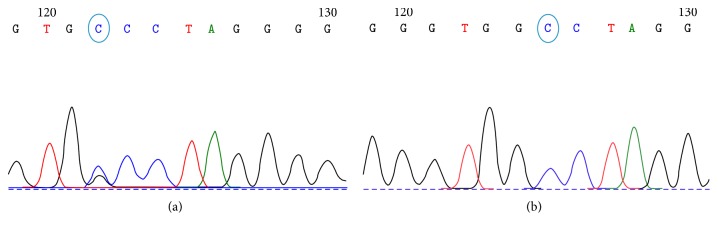
Representation of novel mutation identified at intron 8. (a) Patient genotype (GC) and (b) control genotype (GG).

In the genetic analysis, “C” was identified as a risk allele (*p* = 0.001) from *Fok*I (rs2228570), while the risk genotype was CC (*p* = 0.0015) making the population at risk of T1D development. Allele “T” from *Apa*I (rs7975232) was found to be significant with T1D (*p* = 0.036), but at the genotype level “TT” and “TG,” significant association was detected between T1D and the studied SNP (*p* = 0.014). No significant difference was observed for *Taq*I among patients and controls for allelic and genotypic frequencies (*p* > 0.05). A novel SNP was identified as a result of the sequencing shown in Figure 2 about 62 bp downstream from the *Apa*I polymorphic site and submitted to NCBI (GeneBank Acc number KT280406); the detected mutation was G into C. This novel SNP was only identified in 4.18% of patients while it was not found in the controls.

Protein alignment showed that tryptophan changes into arginine in *FokΙ* with a change in allele whereas no change in amino acid occurred in *Taq*I sites.

Haplotype analysis depicted both the protected and susceptible haplotypes for T1D. For the four genetic polymorphisms, haplotypes CCGC, CCGG, CCTC, and CCTG were significantly associated with T1D (*p* < 0.01). However, the frequency of CTGG and CTTG was higher in controls (0.933, 0.067, resp.) as compared to patients (0.051, 0.000, resp.); so they were protective against T1D development (*p* < 0.01), as shown in Table 3. A correction with eight gives only significance for the CTTG haplotype which was protective against T1D development.

**Figure 3:**
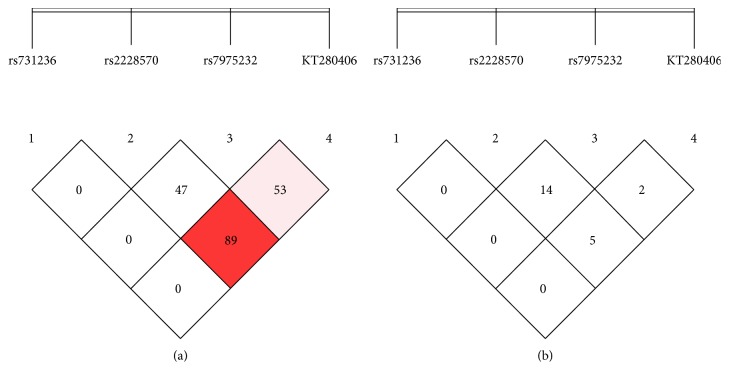
Location and map of linkage disequilibrium (LD) in SNPs at *VDR* gene are presented. The SNP numbers are indicated at the top of haploview. (a) LD = D^/^ and (b) LD coefficient. Red = linkage is highly significant, pink = significant, and white = not significant (decrease in color sharpness indicated reduce chances of disease transfer to next generation).

Linkage disequilibrium (LD) of genetic variants revealed the possible genetic recombinations (D') between the SNPs within loci. Strong LD was described in *VDR* gene (rs2228570, rs7975232, and KT280406) with 89.8% (D^'^ = 0.898) recombination. Correlation between rs7975232, rs2228570, and KT280406 revealed that these SNPs are not a good predictor of each other (*r*^2^ = 0.150) (Figure 3).

## 4. Discussions

The results of the present study revealed the existence of an association between *VDR*-*Fok*I, *VDR*-*Apa*I polymorphisms, and T1D in the Pakistani population. The association of the *VDR* gene polymorphism at four polymorphic sites (*FokΙ*, *Apa*Ι, KT280406, and *Taq*Ι) with T1D was examined in the present study. In the current study, *Apa*I does violate HWE (*p* < 0.05). This might be because Pakistan is a multicultural country with specific traditions which were followed for generations. Among those traditions, one of the most common traditions is intercaste marriages and absence of random mating, ultimately reducing genetic recombination. These also might be the reason for the HWE violation.

The current study reported that *FokΙ* polymorphism significantly (*p* = 0.001) increased the chances of disease development in the Pakistani population. However, the frequency of the *FokΙ* “C” allele was higher in cases in Hungary [18]. The findings on Dalmatian and Japanese populations were in line with the results of our study that the *FokΙ* restriction site was significantly associated with T1D [19, 20]. In contrast with the current results, the findings on two subpopulations of Spain and Iran reported no association of *FokΙ* polymorphism with T1D. Moreover, the frequency of the CT allele was reported to be higher in cases as compared to controls in the Iranian Population [21, 22].

No significant genotypic association was observed between *ApaΙ* polymorphism and T1D in the current study; however (*p* < 0.05), in the Taiwanese population, a significant association was reported between the *ApaΙ* polymorphism and the onset of T1D [23]. In line with the present observation, studies on Greece, Finland, and Norway reported that no association exists between *Apa*Ι polymorphism with the development of T1D [24–26].

The present study demonstrated that the C allele of *Taq*Ι was frequently present in both the case and control, and no association exists between *Taq*Ι polymorphism and T1D development in the Pakistani population. In contrast to our study, it was reported that the T allele was more common in 79.5% of healthy subjects as compared to diseased, and a significant association exists between *Taq*Ι polymorphism and T1D [27]. Genetic heterogeneity was reported to be influenced by the *Taq*Ι polymorphism in Romanian T1D individuals and significantly associated with T1D [20, 28].

The effect of *VDR* gene polymorphism on its functioning has not been completely understood, and it was considered that different SNP interactions lead to the alteration in gene functioning [25]. The current study revealed that the frequency of haplotypes CTGG and CTTG of *Taq*I-*Fok*I-*Apa*I-KT280406 was significant and protects against disease development. CCGG, CCTC, and CCTG were significantly associated (*p* < 0.01) and involved in disease development. In line with current observation, CCG was reported as a significantly associated haplotype in the German population and acts as a protective marker whereas CTG, CTT, and CCT were not associated with the haplotype of T1D [29].

A significant association between rs2228570, rs731236, rs7975232, and rs1544410 and development of T1D was reported in Taiwan, India, Hungary, German, Japan, Croatia, the Netherlands, Chile, and Spain [18, 22, 23, 29–33]. However, in Brazil, Finland, Romania, the United States, and Norway, the population lacks the association of T1D with rs731236, rs2228570, rs7975232, and rs1544410 [17–28, 34, 35].

## 5. Conclusions

Our case-control study demonstrated that *VDR* polymorphism in the *Fok*I, *Apa*I, and KT280406 regions is susceptible to T1D development in the Pakistani population. Further studies should be carried out to determine the role of genetics in diabetes onset so that the susceptible communities can be targeted.

## Supplementary Material

S. Figure 1. Chromatographic representation of rs731236 (TaqΙ) of VDR gene (chromosome 12). (a) Patient genotype (CC) (b) Control genotype (CC). S. Figure 2. Chromatographic representation of rs7975232 (ApaΙ) of VDR gene (chromosome 12). (a) Heterozygous mutation (GT) (b) Homozygous Mutation (TT) (c) Wild Type (GG). S. Figure 3. Chromatographic representation of rs731236 (FokΙ) of VDR gene (chromosome 12). (a) Patient genotype (CC) (b) Wild type genotype (TT). S. Figure 4. Representation of the blast of sequences on NCBI (a) rs731236 (No mutation) (b) rs7975232 (G changes into T) (c) rs10735810 (T changes into C) (d) Novel Mutation (G changes into C).

## Figures and Tables

**Table 1:** Clinical parameters of the individuals.

Parameters	Patient (*n* = 102)		Control (*n* = 100)	
	Male (*n* = 54)	Female (*n* = 48)	Male (*n* = 52)	Female (*n* = 52)
Age (years)	12.5	14.05	13.40	14.13
Age of diagnosis (years)	4.35	5.02	0	0
Positive family history	52%	70%	0%	0%
Physical activity	44.83%	27.3%	100%	100%

**Table 2:** Single site test of genetic variants in T1D and controls.

Regions	SNP	Patients *n* (%)	Controls *n* (%)	*χ* ^2^	*p* values
Exon 2	rs2228570 (*Fok*I)				
	Genotype				
	CC	84 (82.4%)	0 (0.0%)	182.95	0.0016^∗^
	CT	13 (12.7%)	0 (0.0%)		
	TT	5 (4.9%)	100 (100%)		
	Allele				
	C	181 (88.7%)	0 (0%)	321.48	0.0015^∗^
	T	23 (11.3%)	200 (100%)		

Intron 8	rs7975232 (*Apa*I)				
	Genotype				
	GG	33 (32.4%)	86 (86.0%)	64.34	0.014^∗^
	GT	26 (25.5%)	0 (0.0%)		
	TT	43 (42.2%)	14 (14.0%)		
	Allele				
	G	92 (45.1%)	172 (86%)	74.61	0.015^∗^
	T	112 (54.9%)	28 (14%)		

Exon 9	rs731236 (*Taq*I)				
	Genotype				
	CC	204 (100%)	200 (100%)	1.01	0.310
	Allele				
	C	204 (100%)	200 (100%)	1.01	0.496

Intron 8	KT280406				
	Genotype				
	CG	20 (4.1%)	0 (0%)	21.76	0.006^∗^
	GG	184 (95.9%)	30 (100%)		
	Allele				
	C	20 (9.8%)	0 (0%)	20.63	0.026^∗^
	G	184 (90.2)%	200 (100%)		

*χ* ^2^: chi-square test; ∗ represents significance at the 0.05 level.

**Table 3:** Haplotype analysis of the *VDR* gene located on chromosome 12.

Haplotype (rs731236-rs2228570-rs7975232-KT280406)	Case (freq)	Control (freq)	*p* value
CTGG	0.046	0.860	0.001^۞^
CTTG	0.054	0.014	0.004^۞^
CCGC	0.035	0.000	0.007^∗^
CCGG	0.368	0.000	0.002^∗^
CCTC	0.051	0.000	0.001^∗^
CCTG	0.434	0.000	0.006^∗^

∗ represents a significant association of haplotypes with T1D. ^۞^ represents a significant association of haplotypes protective against T1D.
