# Supplementary material for: Vitamin D Receptor Gene Polymorphisms Influence T1D Susceptibility among Pakistanis
Source: Int J Genomics. 2017 Dec 3;2017:4171254. doi: 10.1155/2017/4171254 (PMC5733195; doi:10.1155/2017/4171254)
Supplement: Supplementary file 1 — S. Figure 1. Chromatographic representation of rs731236 (TaqΙ) of VDR gene (chromosome 12). (a) Patient genotype (CC) (b) Control genotype (CC). S. Figure 2. Chromatographic representation of rs7975232 (ApaΙ) of VDR gene (chromosome 12). (a) Heterozygous mutation (GT) (b) Homozygous Mutation (TT) (c) Wild Type (GG). S. Figure 3. Chromatographic representation of rs731236 (FokΙ) of VDR gene (chromosome 12). (a) Patient genotype (CC) (b) Wild type genotype (TT). S. Figure 4. Representation of the blast of sequences on NCBI (a) rs731236 (No mutation) (b) rs7975232 (G changes into T) (c) rs10735810 (T changes into C) (d) Novel Mutation (G changes into C). [file 4171254.f1.docx]

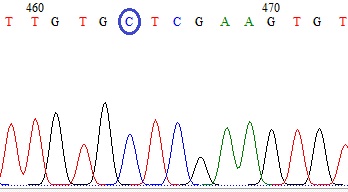

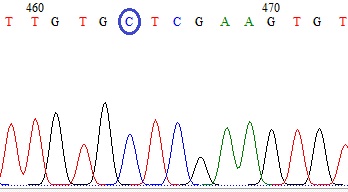


**a**

**b**

S. Figure 1. Chromatographic representation of rs731236 (TaqΙ) of *VDR* gene (chromosome 12). **(a)** Patient genotype (CC) **(b)** Control genotype (CC)


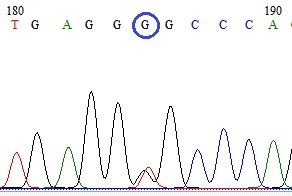

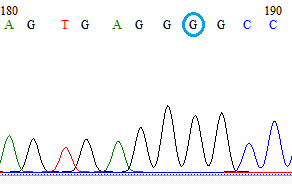

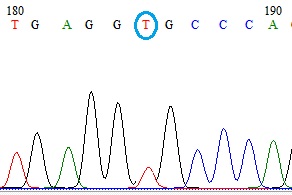


**a**

**b**

**c**

S. Figure 2. Chromatographic representation of rs7975232 (ApaΙ) of *VDR* gene (chromosome 12). **(a)** Heterozygous mutation (GT) **(b)** Homozygous Mutation (TT) **(c)** Wild Type (GG)


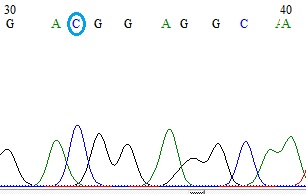

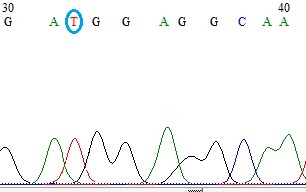


**a**

**b**

S. Figure 3. Chromatographic representation of rs731236 (FokΙ) of *VDR* gene (chromosome 12). **(a)** Patient genotype (CC) **(b)** Wild type genotype (TT).


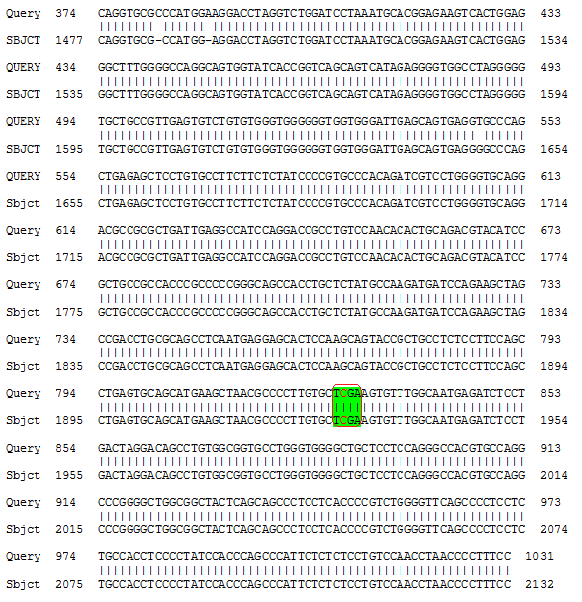
(a)


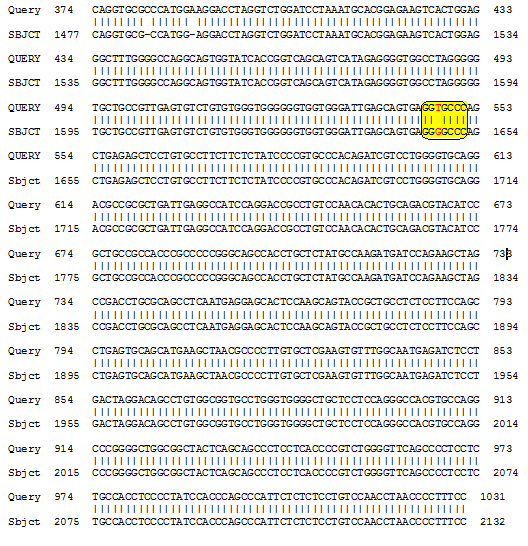
(b)


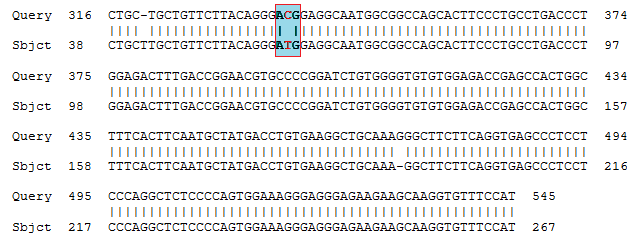


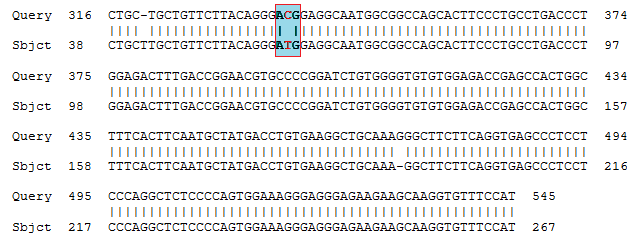
(c)


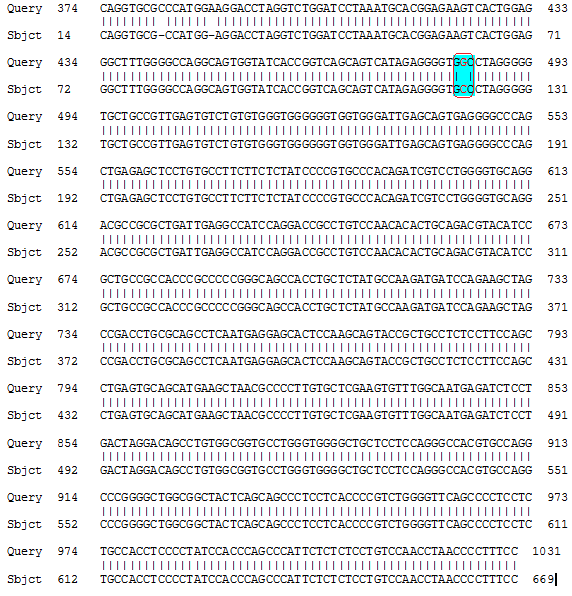


(d)

S. Figure 4. Representation of the blast of sequences on NCBI (a) rs731236 (No mutation) (b) rs7975232 (G changes into T) (c) rs10735810 (T changes into C) (d) Novel Mutation (G changes into C).
